# Evaluating the Efficacy of Online and Blended Learning Training Courses in the Validation Approach

**DOI:** 10.1093/geroni/igaf122.2597

**Published:** 2025-12-31

**Authors:** Vicki de Klerk-Rubin, Harvey Sterns

**Affiliations:** Validation Training Institute, The Hague, Zuid-Holland, Netherlands; The University of Akron, Akron, Ohio, United States

## Abstract

This study examines the effectiveness of online and blended learning training courses using the Validation approach as a psychosocial intervention. It focuses on pre- and post-training assessments conducted through surveys. The research aimed to measure participants’ knowledge, self-efficacy, and perceived stress in relation to the Validation approach, exploring how different training formats influence these factors. Additionally, the study provides insights into the challenges of using digital survey tools and presents significant findings on the impact of this psychosocial intervention on self-efficacy and stress levels among participants.

